# An Animated Meeting of Constituents

**Published:** 1912-12-28

**Authors:** 


### THE METROPOLITAN HOSPITAL SUNDAY FUND.

### An Animated Meeting of Constituents.

### A GRAVE CRISIS AND THE WAY OUT.

The annual general meeting of the constituents of this Fund was held at the Mansion House on Friday, 20th inst., under the chairmanship of Sir John C. Bell, Bart., Vice-Chairman.

The notice convening the meeting was read by the Secretary, Sir Edmund Hay Currie; also the minutes of the last annual meeting, which were approved and signed.

#### The Report of the Council Received.

The Bishop of Willesden proposed that the report of the Council for the year 1912 be and is hereby received. He said the Bishop of London had asked him to explain to the meeting that his absence from it did not mean that his lordship's interest in the Fund had suffered any diminution, but he found his time so fully occupied in connection with Ordination candidates that it was impossible for him to attend. He (the speaker) considered it a great honour to be asked to propose the resolution. It could be safely concluded that the report was published with the greatest accuracy. A satisfactory feature was that the amount which had been collected this year slightly exceeded last year's sum. Unhappily, as one saw by the papers, the amount given to the King's Fund showed a slight falling off. But supporters of the Sunday Fund should not preen themselves too much, because this year's total was not by any means the highest. But the number of congregations contributing was this year the largest yet reached—namely, 2,132, or exactly twice as many as when the Fund was originated forty years ago; yet the amount contributed had not advanced in proportion. In 1905 the collections realised nearly £49,000, whereas this year's figure was £36,000. Next year would be the fortieth of the Fund's existence, and he hoped it would be signalised by a special effort to retrieve past deficiencies. He remembered the Fund being started, and in those early days there was a delightful amount of competition among the various churches in the town in which he lived, and probably all over England, as to which should do the best for the Fund. Though competition was not always commendable, and was not nearly so good as co-operation, he feared that healthy emulation had largely ceased in connection with the Hospital Sunday Fund. He related how the churchwardens of one church would ascertain the amount collected by a neighbouring church, and, if it was more than their own, quietly pay in another half sovereign; and the chagrin with which they discovered that a similar ruse had been practised by the wardens of the other church. He expressed thanks to all those who had so accurately done the work in connection with the Fund.

The Rev. W. Hardy Harwood seconded the resolution. He said that nearly twenty years ago he heard the late Canon Fleming give a description in the Mansion House of his habit of writing letters to every member of his congregation, before Hospital Sunday, who was likely to be absent from church on that day. And he (the speaker) had largely followed that method, and he believed that if it had been done at all generally the fact of the collection-day last year being a very stormy one would not have militated so greatly against the success of the effort, especially as a man would often give, in response to a request, more than he would contribute if he attended. He cordially thanked the press for so generously giving space to advocacy of the Fund, though there was such pressure on their columns, and he hoped that the same kindness would be continued.

#### "The Hospital's!" Statement.

The Rev. L. S. Lewis (St. Mark, Whitechapel) desired, before the resolution was put to the meeting, to ask one or two questions. In spite of what happened, and the discussion which took place at the meeting of constituents last year, St. George's Hospital had received another grant. He reminded the meeting that in 1909 the King's Hospital Fund, which had hitherto given the main grant to St. George's Hospital, withheld it in that year until the hospital authorities satisfied it on two points. It had been found that the hospital had granted a certain portion of the funds contributed for the sick poor to the medical school. Secondly, whereas an agreed system —agreed to by the three great Metropolitan Hospital Funds—of accounts was used by the chief hospitals, St. George's had not used it; consequently a ready comparison of cost and work could not be made with other hospitals. That, as the King's Fund pointed out, was dealing hardly with the charitable public, and was not fair to the other large hospitals, which fell into line in this matter. He referred to a statement published in The Hospital of March 16, 1912, page 596, to the effect that St. George's Hospital in the year 1909 did wrongly pay £875 out of the general funds to medical schools and in a subsequent year a further sum wrongly also. Yet the Sunday Fund came forward and granted that hospital £1,545, thus recouping itself for the loss on the King's Fund. Last year, in consequence, there was a great discussion among the constituents of this Fund, and the Vicar of Whitechapel, the Rev. Mr. Hanks, appealed for peace, because he was most anxious that the public confidence in the Fund should be restored. And Mr. Hanks went on to say that, though an inquiry had been promised before another grant was made, he did not see any evidence that it had been carried out. The Bishop of London also appealed for peace, and on the understanding that the Lord Mayor, Sir Thomas Crosby, would thoroughly thrash the question out before the next meeting of the constituents, which undertaking the Lord Mayor gave, the Rev. Mr. Lewis (the speaker) withdrew his amendment to refer the St. George's grant back, and the original motion was carried. Sir Thomas Crosby must have misunderstood the terms of the undertaking, for the promise had not been specifically kept, although Sir Thomas declared himself satisfied. Sir Edward Fry's Commission reported that hospitals and schools balanced the benefit that each received from the other, and every one saw that it was necessary to pay for the needed examinations of sputum and excretions of patients, but the point was that the King's Fund found that the sum put down for that purpose in the accounts of St. George's Hospital was enormously in excess of that charged in any other hospital, and no less an authority than Sir William Church found it was 180 per cent. compared with payments by other hospitals to their laboratories. Yet they of the Sunday Fund were asked to come and flout the King's Fund by making this grant to that hospital. In Sir Thomas Crosby's report occurred these words: ". . . besides the care of the museum which is the property of the hospital." That was simply part of the balance of the benefit which the hospital got from the school and the benefit which the school obtained from the hospital. Sir Thomas went on to say in his report that the amount expended upon it was in no way excessive, and did not represent a larger cost per occupied bed than was the case at many other London hospitals, although as the accounts of these departments were not kept in a corresponding manner at different hospitals, various items being included or excluded, it was difficult to show that in exact figures. Of course, it was open, said Mr. Lewis, for any hospital to refuse to adopt the suggested system of accounts, and similarly it was open for a fund to refuse to make its grant to a hospital. In the passages of the report which he had touched on he contended that Sir Thomas had condemned St. George's Hospital, which was the only great hospital that had behaved in that manner. St. George's Hospital had been found to be guilty of extravagance, of using money for the school which had been contributed for the benefit of the sick poor. It was said that the protest of constituents at the meeting in December was useless, as the apportionment of grants was made in August. Such a state of things could not be regarded as satisfactory, and its parallel would not be found in the whole of Europe. Moreover, it had been stated by an official of the Hospital Sunday Fund that that Fund did not, like the King's Fund, go into details of management before making a grant, and it did not withhold a grant unless there was a scandal or definite trouble. If that were the case, the constituents should make "definite trouble."

The resolution was then put and carried.

Sir William S. Church, K.C.B., M.D., moved: That the Council for the year 1912 be re-elected for 1913, with the addition of the Rev. the Master of the Temple (Dr. Woods), Adrian Pollock, Esq. (City Chamberlain), the Hon. Frederick Ponsonby, E. M. Harvey, Esq., and Charles Evan Spicer, Esq. Those names were, he said, so well known in the City of London and among the constituents of the Fund that it was unnecessary to do more than propose the resolution.

Mr. John Henry Buxton seconded, and it was carried.

Sir Ernest Tritton, Bart., proposed: "That May 25 be fixed for Hospital Sunday in 1913, and that the cordial co-operation of all ministers of religion within the Metropolitan area be again invited in the usual way." He expressed the earnest hope that the weather would be kinder on the appointed day than last time.

The Hon. Sydney Holland seconded, and it was carried.

### Mr. Lewis's Lost Notices of Motion.

The Rev. L. S. Lewis expressed his great regret that, owing to the serious illness of the Secretary of the Fund, Sir Edmund Currie, a letter which he sent on December 22 last, giving notice of three motions which he intended to move at this meeting, had been lost, he was sure through a pure accident. He regretted that they were not printed for this meeting, and he wished to know whether he would be allowed to bring them forward now.

The Chairman replied that he thought Mr. Lewis had cause for complaint, and he understood that the letter referred to had not only been mislaid but absolutely lost sight of. He (Sir John) was helpless in the matter, as he was bound by the constitution of the Fund. But if Mr. Lewis would send a notice of the resolutions which he wished discussed an opportunity would be given for such discussion. ("When?") The meeting had listened with great patience and at considerable length to the Rev. Mr. Lewis to-day, and he asked all to believe that they of the Council were sincere in their desire to make the Hospital Sunday Fund the success which it ought to be, to the benefit of the necessitous poor. The Council were not paid servants, but were there to do the best they could in the interests of charity. One remark made by Mr. Lewis led him to say that—namely, that investigation was not made into the claims for grants.

The Rev. L. S. Lewis thought that, as the notices of motion were not lost through any fault of his, he should be allowed to proceed with the purpose which he intended to carry out. He asked whether a special meeting would be called to consider the question.

The Chairman said he was anxious to give Mr. Lewis as much licence as possible, and if he should succeed in carrying what he wished now, he (Sir John) would convene a meeting on the matter between now and the meeting of constituents next year. But he had not the power now seriously to put such resolutions. The constitution would not permit of it.

The Rev. L. S. Lewis asked whether such meeting could be promised within two months from now.

The Chairman replied that he could not give any such promise; moreover, it was necessary that the constituents should be apprised of the special meeting.

### A Dangerous Step.

The Vicar of Whitechapel (Rev. G. M. Hanks) said the present situation on this subject was tenfold more difficult than it was last year when that matter was raised. The Fund at the present moment was in danger of taking a step which was calculated to do it grievous harm. Yet he would do his utmost to spare Sir Edmund Currie pain, so great was his affection for the Secretary. Therefore he would make a direct and personal appeal to that gentleman to step in at this juncture and save the Fund from a calamity.

The Chairman assured the meeting that the only object the Council had was to procure the success of the Fund —("That's no answer")—and if gentlemen would display a little more Christian spirit they would help the Fund instead of damaging it.

Mr. Fitzgibbon said that, unless some improvement were introduced into the management of the Fund, the whole thing was liable to be ruined. There had been promises of amendment, but such had not been carried out, yet one would have concluded that the Council had been considering the matter all this time.

The Chairman said he had nothing to add, but wished to thank those present for their attendance at the meeting, and he hoped that the spirit which he had foreshadowed would be carried out. (Signs of dissatisfaction.)

### An Appeal to the Chair.

The Rev. W. P. Cromie (Harlesden) made an earnest appeal to the Chairman before he vacated the chair. He came prepared to support the Council of the Fund through thick and thin, but, unless some very definite steps were taken, it would not be possible confidently to count on the support of many of his brethren throughout the diocese. There was a widespread feeling among clergy and laity that this Fund was not managed as it ought to be, and he earnestly hoped that some steps might be taken this afternoon whereby the feelings expressed by Mr. Lewis would receive careful and respectful attention.

The Rev. Scott Lidgett joined in the appeal to the chair, not on the merits of the case, but after the promise given a year ago, and after the lamentable loss of Mr. Lewis's notice of motion he could not help submitting that the Chairman should allow the notices of motion and find out whether they received support at an early meeting—not necessarily within two months, but at an early meeting—for it would be in the interests of that Christian peace which the Chairman had asked for.

Sir Henry Burdett's Suggestion.

Sir Henry Burdett, K.C.B., said he believed he was one of the oldest members, if not indeed the oldest member, of this Council, and he ventured to join in the Rev. Scott Lidgett's appeal to the chair. This was the sixth consecutive year that the constituents of this Fund had not enjoyed the full rights belonging to them, owing to the raising of technicalities, but which rights must be conserved if the Fund was to live, for at the present time it was not living, but only existing. He wanted to see life infused into the Fund, and he suggested, as a way out, that the Council should consent to examine and report upon Mr. Lewis's resolutions, whatever they might be, which had been lost through the ill-health of the Secretary, and then summon a special meeting of the constituents to deal with their report thereon in due course. That would give a full opportunity for ascertaining the views of the constituents and of the churches of all denominations behind this Fund. Thus harmony might speedily be restored with, he hoped, a good prospect of seeing his ambition fulfilled—namely, that the Fund should raise in one year £100,000. It could be done, and it ought to be done. And the differences which had arisen to-day constituted the very occasion which should be thankfully grasped, and by co-operation and that Christian spirit which had been invoked by the Chairman—for surely in a Hospital Sunday Fund that spirit should be manifest—unite in the great work of the Fund, and go forward and do it. (Applause.)

A Constituent: Will you accept a motion for the adjournment of this meeting?

The Chairman: Certainly not; the meeting is already concluded.

The Rev. Alan Ewbank said he came specially to support the Fund, but the present proceeding was the most extraordinarily unfair thing he had ever faced, and he could not throw in his sympathies with it. He did not judge on the merits of the difficulty which had arisen, but when a difficulty was brought up it should be faced fairly, not burked.

The Hon. Sydney Holland agreed that nothing could have been more unfortunate than the loss of Mr. Lewis's resolutions on account of the Secretary's illness, but the Council were in the difficulty that they could not bring forward a resolution at that meeting unless its terms had been sent out to all the constituents. The loss was discovered last Wednesday (two days ago) and it was impossible to get out such notice in time for this meeting. The action of the Council must be governed by the constitution. He was eager to debate in front of the constituents of the Fund Mr. Lewis's proposed alterations, and if Mr. Lewis were to carry them—which he very much doubted—it would be a very good reason for calling another meeting, *ad hoc*, to discuss it. But if, on the contrary, at this meeting Mr. Lewis found he had considerable support he should bring it forward at the next annual meeting of constituents, and sc on from year to year until he got a redress of his grievances. He was smarting for the opportunity of meeting those who were abusing the Fund with an ignorance which showed they had not gone properly into the matter. Therefore it was not cowardice which dictated the decision of the Chairman, but the constitution of the Fund.

The Chairman said he saw no objection to the meeting taking Mr. Lewis's resolutions and discussing them as long as it wished, and if they were carried, he would call a meeting to discuss what had been carried. But if, after hearing both sides, they were not carried, then Mr. Lewis must accept defeat. He personally had no objection to staying there until eight or nine o'clock to hear the arguments, but it could not become part of the official proceedings of the day.

**"The Terms of the Constitution."**

The Rev. L. S. Lewis asked the Chairman to read to the meeting the exact terms of the law of the constitution which made it impossible to have these resolutions legally put.

The Chairman said the meeting was officially closed, but Mr. Lewis could address the meeting, and if he succeeded in carrying the resolutions a meeting would be called at a future date to officially discuss them.

The Rev. L. S. Lewis retorted that he would not trouble the meeting until the Council thought it consonant with their dignity to hear a notice of motion which had been duly sent in.

The Bishop of Willesden appealed to Mr. Lewis to seize the opportunity of ventilating the matter, after which the question could be sent broadcast among the constituents and a decision come to.

The Rev. L. S. Lewis said the meeting was now much thinned, and he would wait until the Council could summon a meeting when the constituents would have their chance.

Counsel's Opinion.

The Hon. Sydney Holland read the opinion of counsel on the matter, which said: "I feel grave doubt as to the competency of a meeting of the constituents to effect so drastic a change of the constitution as is suggested by Mr. Lewis. But even assuming that a meeting of the constituents is competent to do so, I feel clear that a special notice of a proposed motion for making the alteration ought to be given to the constituents before the meeting is held at which it is proposed to bring on the motion. In these circumstances I am of opinion that Mr. Lewis's motion should not be put by the Chairman, and, indeed, that the Chairman should not allow Mr. Lewis to move it."

Question of a Special Meeting Referred to the Council.

The Rev. L. S. Lewis said all that was asked for was a properly summoned meeting at an early date; it would be preferable to a snatch vote now.

The Rev. W. P. Cromie said some of the lukewarmness in regard to the Fund was traceable to the feeling on this matter, for it was felt by many that here was a definite grievance. He therefore asked Mr. Lewis to let the matter drop now, conscious that the Council would, by the course they would take in the days to come, give tho public eevry confidence in the working of the Fund.

The Rev. L. S. Lewis said that was the assurance which had been asked for for a long time.

As Sir John Bell was in the act of vacating the chair a member of Council proposed a vote of thanks to him, and the meeting dispersed.

(*Continued on p.* 360.)

### Rev. L. S. Lewis's Resolutions.

Few or none of those who attended the meeting of constituents of the Hospital Sunday Fund at the Mansion House on the 20th inst., except the mover, knew the nature of the resolutions which had been lost by the Secretary. It may be interesting, therefore, to reproduce the following explanation and copy of the resolutions signed by Mr. Lewis which were published in the *Daily Telegraph* of the 19th inst :

To the Editor of "The Daily Telegraph."

Sir,—May I ask the hospitality of your columns? By an unfortunate accident, which has been explained to me, three important resolutions of mine, to be moved at next Friday's meeting of the above Fund at the Mansion House, at 3 p.m., were omitted to be printed on the agenda-paper. These resolutions affect the future of the Fund.

The situation is as follows: By the laws of the Fund each contributing congregation is supposed to have a voice in the management of the Fund through its minister and two laymen. They are called together once a year at the annual meeting. They do not elect the Distribution Committee. When they arrive they are asked to approve of grants of money which have been already paid out months before. It is a farce. The grants are made in August. The meeting has, by the laws, to be in December. It has actually happened that exception has been taken to a grant, and when an amendment has been brought forward to approve of the report and awards as a whole, but to refer that particular grant back for reconsideration, the constituents have been told that it is of no use, that the money has been actually paid. In other words, the presence of the constituents there is a sham calculated to lure the public into a belief that there is public control of the management of the Fund. I do not think that anyone can be found to defend laws compelling so ridiculous and grotesque an action, and making fools of everyone who solemnly goes to the meeting.

I am therefore moving:

1. That Law III. of the constitution shall be changed to the following:

III.—The Committee of Distribution shall consist of the Lord Mayor as president and the hon. secretaries, *ex officio*, and nine other members (of whom four only shall be doctors or surgeons), who shall be elected at the annual general meeting.

2. That Law X. of the constitution shall be changed to the following:

X.—The Committee of Distribution shall present their report to the Council for approval, and before the Fund shall be distributed the Council's recommendation shall be confirmed by the annual meeting. In the event of any portion of the award being rejected at that annual meeting, an arbitration committee, whose decision shall be final, shall be appointed at that meeting to deal with that particular point; such arbitration committee to consist of three members elected by the constituents of the Fund at that meeting, three members elected by the Council, and a chairman chosen from outside the Fund to be agreed upon by the other six members of the arbitration committee.

3. That in Law I. of the constitution the words "in the month of December" be deleted.

Your insertion of this letter will give a chance to those who favour the old order (if such people can be found) and those who favour the new, or rather some new order, to consider the proposed alterations in the laws of this important Fund.—Your obedient servant,

Lionel S. Lewis.

St. Mark's Vicarage, Whitechapel, E., December 18.

